# The negative impact of the COVID‐19 pandemic on UK haematology registrars’ well‐being and training: Results of a UK nationwide survey

**DOI:** 10.1002/jha2.279

**Published:** 2021-08-26

**Authors:** Mohammed OA Altohami, Alexander Langridge, Enas MK Shareef, Phillip LR Nicolson

**Affiliations:** ^1^ University Hospitals of Leicester NHS Foundation Trust Leicester Leicestershire UK; ^2^ HaemSTAR Birmingham UK; ^3^ Newcastle Upon Tyne Hospitals NHS Foundation Trust Newcastle Upon Tyne UK; ^4^ Department of Physiology College of Medicine National Ribat University Khartoum Sudan; ^5^ College of Medical and Dental Sciences Institute of Cardiovascular Sciences University of Birmingham Birmingham UK; ^6^ University Hospitals Birmingham NHS Foundation Trust Birmingham UK

**Keywords:** COVID19, haematology training, postgraduate medical education

## Abstract

The COVID‐19 pandemic has disturbed medical training. Haematology registrars were surveyed using SurveyMonkey. Eighty‐nine out of 269 (24.9%) responded. Reported stressors included concerns about transmitting the infection, disruption of leave, inferior patient outcomes, survivors’ guilt and interruption of career progression. Only 31.2% felt ready to progress to the next training stage. Reported causes of lack of training were disruption of clinics and training days and utilisation of telephone consultations. Several stressors negatively impacted haematology registrars’ well‐being, training and progression. More emphasis on psychological support, outpatient clinic work and e‐learning is needed.

## 1 INTRODUCTION

The COVID‐19 pandemic first started in China in December 2019 and subsequently rapidly spread worldwide. The multidimensional impacts of the ongoing pandemic include disturbance to all aspects of postgraduate medical education [1, 2, 3].

The competence‐based haematology specialist training in the United Kingdom encompasses both clinical and laboratory training. The rigorous assessment of trainees’ competence progression across various curricular areas is aided by consultant reports and performance in workplace‐based assessments (WPBAs). Passing all parts of the FRCPath examinations is mandatory. There is no published research on the impact of the COVID‐19 pandemic on UK haematology registrar training. Our aim is that this work helps all stakeholders to make plans to mitigate the negative impacts on training of current and future pandemics.

## 2 METHODS

A questionnaire (reproduced in the Supporting Information) was designed using SurveyMonkey® (San Mateo, CA) and was distributed by the HaemSTAR network [4]. Responses were collected between June and October 2020. The statistical package for social sciences version 17 (SPSS‐17) (Chicago, IL) was used for data analysis. Respondents’ opinions were gathered using Likert items and described using median and inter‐quartile range (IQR). A median of 4 or more was assigned to determine respondents’ agreement regarding a statement, while a median of 2 or less was assigned to indicate disagreement. Nonparametric tests were applied using two‐tailed testing and α value of 0.05.

## 3 RESULTS

The survey was sent to 269 haematology registrars, and 89 completed responses were received (response rate of 24.9%); see Supplementary Figure 1. The median age for respondents was 34 years (IQR: 31–36) and 56 (62.9%) were females. Six (6.7%) had active and ten (11.2%) had historic mental health issues.

Stressors affecting the respondents during the pandemic are shown in Table 1. Concerns about transmitting the infection to household contacts, disruption of travel or leave plans and lockdown measures received the highest rating as stressors, as evident by a median and lower limit of IQRs of 4. Reported stressors were fear of infection, care needs including childcare, inferior patient outcomes due to COVID‐19 infection or to modifications of haematological management, interruption of career progression (including inability to achieve learning objectives or required WPBAs), interruption of public transport used for personal activities and feeling safer in haematology compared to frontline staff (survivors’ guilt). Fear of adverse pregnancy outcome, the need to self‐isolate, staff‐shortage and the cancellation of out‐of‐program experiences were also described.

**TABLE 1:** Respondents’ perceptions towards various stressors during the pandemic response period reported as the median and the IQR for nineteen 5‐point Likert items

Item	Median	IQR	*p*
Lack of appropriate PPE	3	2–4	<0.001
Inferior patients’ outcomes due directly to COVID (i.e. infection)	4	3–4	<0.001
Inferior patients’ outcomes due indirectly to COVID (e.g. adoption of non‐intensive management strategies)	4	3–4	<0.001
Redeployment to non‐familiar areas and other specialties	2	2–4	<0.001
Feeling guilty being safer in haematology compared to my colleagues on the ‘frontline’	4	2.25–4	<0.001
Feeling guilty about shielding due to underlying health issues	3	2–3	0.001
Increased haematology workload	3	2–4	0.005
Interruption of career progression including inability to meet learning objectives outlined in the personal development plan (PDP) or inability to collect required work‐place‐based assessments	4	3–4.25	<0.001
Interruption of research projects	3	3–5	0.01
Interruption of/changes in public transport used to get to and from work	3	2–4	0.251
Interruption of/changes in public transport used for personal activities such as shopping, leading to implications on free time	4	3–4	<0.001
Public health and government interventions including mobility restrictions and social isolation measures	4	4–4	<0.001
Disruption of leave or travel plans	4	4–5	<0.001
Care needs including childcare	4	3–5	0.01
Fear of infection for yourself or your immediate contacts	4	3.75–5	<0.001
Concerns about transmitting infection to co‐habitants living in the same house as me	4	4–5	<0.001
Loss of income e.g. locum opportunities	2	2–3	<0.001
Bereavement or infection due to COVID affecting colleagues, friends or family members	2.5	2–4	0.103
Spiritual stresses e.g. suspension of prayers and closure of places of worship, and pressure to trim your beard grown for religious purposes	3	2–3	0.02

Respondents did not agree that bereavement related to COVID‐19, loss of income or redeployment to other clinical areas caused stress. Perceptions of redeployment as a source of stress, however, varied significantly between deaneries (*p* = 0.007). Respondents were asked whether the training they had received to enable safe redeployment was adequate. Forty‐eight out of 79 (60.8%) had not been redeployed. Of the remaining 31, only six (19.4%) thought their redeployment training was adequate. The degree of satisfaction with training delivered to enable safe redeployment was significantly different between deaneries (*p* = 0.027). Respondents reported that redeployment negatively impacted training, particularly morphology training and preparation for exams.

Fourteen respondents (17.5%) reported infection with SARS‐CoV‐2. The infection rate was significantly different between deaneries (*p* = 0.003). Twenty‐two (27.5%) reported infection in a spouse or relative. Negative impact on psychological and mental well‐being was reported by 38 (47.5%) respondents. This was significantly more in females (OR 5.74, *p* = 0.001). On a 10‐point scale from −5 to +5, respondents subjectively felt their overall level of stress has increased to +1.74 ± 0.24 (SD 2.16) compared to pre‐pandemic time.

Respondents strongly agreed that the impact of the pandemic on the promotion of their overall professional skills was negative (median 4, IQR 3–4, *p* = 0.005). However, their perceptions on the impact of the pandemic on the progression of specific skills; inter‐personal, communication, management, administrative, diagnostic and clinical haematology skills were all neutral (see Supplementary Figure 2).

Only 24 respondents (31.2%) thought they had acquired all training needed to progress to the next stage. Thirty‐five (45.5%) thought they should be allowed to progress with mitigating measures to replace lost training. Ten (13%) felt they should receive extension of training.

Respondents thought this lack of attainment of required training was due to cancellation of didactic teaching sessions, loss of exposure in the outpatient clinic settings and increased utilisation of telephone consultations (see Table 2). The negative impact caused by cancellation of teaching was significantly different between deaneries (*p* = 0.018). Respondents thought locally arranged virtual training sessions were effective and informative. Eleven respondents (14.3%) had not had such sessions.

**TABLE 2:** Respondents’ perceptions on the reasons underlying the lack of training progress during the pandemic

Item	Median	IQR	*p*
Loss of training opportunities in the inpatient setting	3	2–4	<0.001
Loss of training opportunities in the outpatients’ clinics setting	4	4–5	<0.001
Loss of training opportunities in the haematology day unit setting	3	2–4	0.252
The utilization of telephone clinics in preference to face‐to‐face clinics	4	3–4	<0.001
Direct or indirect involvement in the care of patients with COVID‐19	3	3–4	<0.001
Cancellation of didactic teaching and training days	5	4–5	<0.001

Respondents thought more investment in virtual learning and virtual training days would help replacing lost training opportunities (see Supplementary Table 1).

## 4 DISCUSSION

Our data reveal that, among our respondents, the COVID‐19 pandemic was associated with multiple psychosocial and work‐related stressors, negative physical and mental health impact, poor academic achievement and skills progression, loss of training opportunities (especially in the outpatient clinic and laboratory settings), loss of teaching sessions and lack of readiness to progress to the next stage. There was no agreement on which professional domain was particularly affected. This likely reflects an overwhelming sense of lack of progression, differing learning needs among respondents according to their level of training and the success of mitigating measures introduced by their training sites.

Fear of transmitting infection to immediate contacts and/or patients had been also reported in the flu pandemic [5]. Lack of appropriate personal protective equipment (PPE) was not reported as a stressor by our respondents despite massive media attention at the beginning of the pandemic. This might be due to the timing of data collection which coincided with the end of the first wave when PPE supplies were more secure. More rigorous infection control practices on haematology wards including routine screening of patients and staff and lower prevalence of aerosol generating procedures are other possible explanations.

Our study showed a high prevalence of negative psychological impact. A previous systematic review showed increased adverse mental health outcomes among healthcare workers (HCWs) during COVID‐19, especially if they were in the frontline, working in high risk areas or redeployed to such areas [6]. Distress, depression, anxiety and insomnia affected 30–70% of HCWs according to a study from China [7]. Psychiatric illness caused the loss of 475,000 full‐time equivalent days representing 31.8% of all NHS staff absences in June 2020 [8]. This is a 24% increase from pre‐pandemic levels [9]. These data indicate that more psychological support is needed.

17.5% of our respondents had SARS‐CoV‐2 infection, which is much higher than the prevalence of positive antibody tests of 5.6% and 4.2% in England and Wales, respectively, in September 2020 [10], but similar to the rates of infection in HCWs reported in COVID‐19 (19%) [11] and during the Middle East Respiratory Syndrome (MERS) outbreak (19.9%) [12]. Sickness absence due to COVID‐19 infection was responsible for nearly one third of all NHS staff sickness absence in April 2020 [8].

Our respondents felt that they suffered due to high rates of redeployment and poor preparation to allow this to happen safely. This would raise concerns regarding safety and efficiency, loss of training opportunities, the impact on haematology staffing levels and the risk of cross‐contaminating haematology units.

Less than a third of participants thought they had achieved the training required to progress to the next stage. This is much lower than the rate of satisfactory outcome among haematology trainees in 2019 (55%) [13]. The major disruptions of training revealed by our data are in line with a recent General Medical Council survey where more than two thirds reported disruption of training [14]. Reported causes for this lack of achievement were cancellation of didactic teaching sessions, reduced laboratory experience, changes to outpatient clinics and adoption of telephone consultations in preference to face‐to‐face consultations. Outpatient clinics and laboratory responsibilities represent the majority of haematologists’ clinical work. We thus recommend that deaneries, as part of their plans to mitigate the effects of the pandemic, put greater emphasis on allowing registrars to have more access to outpatient clinics and laboratory work. This is especially true as we predict the utilisation of telephone clinics is likely to persist for prolonged periods. Our study shows a need to develop and use local and national virtual morphology training platforms [15]. UK NEQAS EQATE is one such platform [16]. Many UK laboratories are starting to use haematology analysers with image analysis capabilities (e.g. CellaVision®). This may facilitate remote morphology reporting in‐ and out‐of‐hours. This together with other changes to facilitate remote working (e.g. changing bleep systems and allowing remote access to hospital systems) would allow shielding registrars (e.g. due to pregnancy) to remotely support their onsite colleagues. Remote working would also facilitate social distancing and minimise in‐hospital infection transmission at peak epidemic transmission times. Many of the teaching sessions and courses are targeted to exam preparation. This might explain the perception that their interruption contributed to the lack of satisfactory training. We are unable to assess whether trainees at different stages of training (i.e. pre‐ or post‐exams) were differently impacted by cancellation of teaching. E‐learning was recommended to replace lost teaching.

In summary, despite multiple studies examining the impact of COVID‐19 on medical school education [17] this is the first published survey to report the negative impact of the COVID‐19 pandemic on haematology registrars. The pandemic and its response were associated with many stressors, including negative impact on physical and mental health, poor academic achievement and lack of readiness to progress to the next stage among haematology trainees. Increased emphasis on psychological support, outpatient clinic and laboratory experience and on e‐learning are urgently required to compensate for the lost training and to mitigate the future negative effects of COVID‐19 as it becomes endemic in society.

## CONFLICT OF INTEREST

MOAA has received support to attend a conference from Janssen. PLRN has received research grants from Novartis, Rigel and Principia Biopharma as well as speaker fees from Bayer, Grifols and Takeda. AL and ES have no conflicts of interest to declare.

## AUTHORS CONTRIBUTION

MOAA proposed the study, drafted the questionnaire, analysed the data and drafted the manuscript. PLRN, ES and AL reviewed and edited the proposal, questionnaire and the manuscript.

## Supporting information

Supporting information
