# Supplementary material for: The negative impact of the COVID‐19 pandemic on UK haematology registrars’ well‐being and training: Results of a UK nationwide survey
Source: EJHaem. 2021 Aug 26;2(4):785–8. doi: 10.1002/jha2.279 (PMC8657531; doi:10.1002/jha2.279)
Supplement: Supplementary file 1 — Supporting information [file JHA2-2-785-s001.docx]

Supplementary Figure 1: Survey response rate in different UK regions and overall.

Supplementary Figure 2: Respondents were asked for their degree of agreement on whether the pandemic had had a positive impact on particular professional skills. The number of responses for each 5-part Likert item is shown on the *x* axis.

| Item | Median | IQR | p |
| --- | --- | --- | --- |
| More investment in virtual learning platforms including virtual training days and conferences | 4 | 4 - 5 | < 0.001 |
| More investment on online learning (e-learning) including pre-recorded videos and interactive Continuous Medical Education (CME) cases | 4 | 4 - 5 | < 0.001 |
| Make the core training (currently Internal Medicine Training [IMT]) 3 years for prospective haematology applicants | 3 | 2 - 3 | < 0.001 |
| Dedicate time for general medicine and Intensive Care Unit (ICU)/High Dependency Unit (HDU) training or allow dual accreditation with General Internal Medicine (GIM) for haematology trainees | 3 | 1 - 3 | < 0.001 |

Supplementary Table 1: Respondents’ perceptions on the potential benefit of different possible remedial actions.
